# Technical note: Consistency of IAEA's TRS‐483 and AAPM's extended TG‐51 protocols for clinical reference dosimetry of the CyberKnife M6 machine

**DOI:** 10.1002/acm2.13976

**Published:** 2023-03-30

**Authors:** Jasmine Duchaine, Daniel Markel, Jean‐Luc Ley, Dominique Béliveau‐Nadeau, Karim Zerouali, Robert Doucet, Hugo Bouchard

**Affiliations:** ^1^ Département de physique Université de Montréal, Campus MIL Montréal Québec Canada; ^2^ Centre de recherche du Centre hospitalier de l'Université de Montréal Montréal Québec Canada; ^3^ Département de radio‐oncologie Centre hospitalier de l'Université de Montréal Montréal Québec Canada

**Keywords:** CyberKnife, reference dosimetry, TG‐51, TRS‐483

## Abstract

**Background:**

While IAEA's TRS‐483 code of practice is adapted for the calibration of CyberKnife machines, AAPM's TG‐51 is still the protocol recommended by the manufacturer for their calibration. The differences between both protocols could lead to differences in absorbed dose to water during the calibration process.

**Purpose:**

The aims of this work are to evaluate the difference resulting from the application of TG‐51 (including the manufacturer's adaptations) and TRS‐483 in terms of absorbed dose to water for a CyberKnife M6, and to evaluate the consistency of TRS‐483.

**Methods:**

Measurements are performed on a CyberKnife M6 unit under machine‐specific reference conditions using a calibrated Exradin A12 ionization chamber. Monte Carlo (MC) simulations are performed to estimate $k_{Q_{\mathrm{msr}},Q_{0}}^{f_{\mathrm{msr}},f_{\mathrm{ref}}}$ and $k_{\text{vol}}$ using a fully modeled detector and an optimized CyberKnife M6 beam model. The latter is also estimated experimentally. Differences between the adapted TG‐51 and TRS‐483 protocols are identified and their impact is quantified.

**Results:**

When using an in‐house experimentally‐evaluated volume averaging correction factor, a difference of 0.11% in terms of absorbed dose to water per monitor unit is observed when applying both protocols. This disparity is solely associated to the difference in beam quality correction factor. If a generic volume averaging correction factor is used during the application of TRS‐483, the difference in calibration increases to 0.14%. In both cases, the disparity is not statistically significant according to TRS‐483's reported uncertainties on their beam quality correction factor (i.e., 1%). MC results lead to $k_{Q_{\mathrm{msr}},Q_{0}}^{f_{\mathrm{msr}},f_{\mathrm{ref}}}=1.0004\pm 0.0002$ and $k_{\text{vol}}=1.0072\pm 0.0009$. Results illustrate that the generic beam quality correction factor provided in the TRS‐483 might be overestimated by 0.36% compared to our specific model and that this overestimation could be due to the volume averaging component.

**Conclusions:**

For clinical reference dosimetry of the CyberKnife M6, the application of TRS‐483 is found to be consistent with TG‐51.

## 1 INTRODUCTION

Reference dosimetry protocols assure consistent calibration of external radiotherapy units. The Task Group (TG) 51 of the American Association of Physicists in Medicine (AAPM)^[^ ^1^ ^]^ and its addendum^[^ ^2^ ^]^ provide a simple procedure for the calibration of medical linear accelerators (linacs). The Technical Report Series (TRS) 483^[^ ^3^ ^]^ of the joint working group between the International Atomic Energy Agency (IAEA) and the AAPM, which is based on the formalism of Alfonso et al.,^[^ ^4^ ^]^ provides recommendations for clinical reference and relative dosimetry of small static photon fields used in external radiotherapy with nominal energies ⩽ 10 MV. This code of practice introduces new reference conditions, called machine‐specific reference (msr) conditions, for radiotherapy treatment units which cannot establish conventional reference conditions, and new quality correction factors to correct the calibration coefficient for the difference in chamber dose response due to change in beam quality.

The CyberKnife M6 system (Accuray Inc., Sunnyvale, California, USA) is a specialized device which delivers advanced radiotherapy treatments. Its linac produces a flattening‐filter free (FFF) beam of 6 MV nominal energy. It produces circular field sizes with nominal diameters ranging from 0.5 to 6 cm at 80 cm from the source. While this type of unit cannot establish conventional reference conditions, to this date, the manufacturer recommends the use of TG‐51 for its calibration. Since TG‐51's recommendations cannot be directly applied, the manufacturer proposes certain adaptations. TRS‐483's recommendations are especially adapted for devices such as the CyberKnife and its implementation could lead to differences in absorbed dose to water per monitor unit (MU) during the calibration process when compared to TG‐51.

Some studies^[^ ^5^, ^6^, ^7^, ^8^ ^]^ compared the TRS‐483 protocol with other established protocols in terms of absorbed dose to water per MU. Huq et al.^[^ ^5^ ^]^ compared results of absorbed dose per MU when applying the TG‐51, its addendum and the TRS‐483 to a Varian TrueBeamTM STx linac. Different beam energies, including with flattening filter (WFF) and FFF beams, and different chambers were considered. In all cases, an impact ⩽ 0.3% is observed. However, this study was performed using a linac which can establish conventional reference conditions, unlike the linac considered in this study. Lopes et al.^[^ ^6^ ^]^ compared quality correction factors when applying the Accuray recommendations (TRS‐398^[^ ^9^ ^]^) and the TRS‐483 protocol to a helical tomotherapy unit. Quality correction factors agreed within 1%. Lee et al.^[^ ^7^ ^]^ compared the absorbed dose per MU when applying the TG‐51, its addendum, and the TRS‐483 to a TrueBeam STx 6 MV FFF linac. The authors observed a difference of 0.25% in dose output. Once again, the conventional reference field size could be established by the considered linac in this study. Buchegger et al.^[^ ^8^ ^]^ compared the absorbed dose per MU when applying the TRS‐398 and the TRS‐483 to a CyberKnife unit. A difference of 0.28% in absorbed dose was observed. Their difference is due, in part, to the difference in volume averaging correction factors.

The primary aim of this work is to evaluate the differences resulting from the application of AAPM's TG‐51 protocol for clinical reference dosimetry (including the manufacturer's adaptations) and IAEA's TRS‐483 code of practice for small field dosimetry in terms of absorbed dose to water per MU for a CyberKnife M6. The secondary aim is to evaluate the consistency of TRS‐483 for different situations of its application, that is, when using an experimentally evaluated in‐house volume averaging correction factor, a generic beam quality correction factor provided in the TRS‐483, and an in‐house Monte Carlo (MC) evaluated beam quality correction factor.

## 2 MATERIALS AND METHODS

### 2.1 Protocols overview

Both AAPM's TG‐51 and IAEA's TRS‐483 protocols are based on the use of a calibrated cylindrical ionization chamber whose calibration is traceable to a primary standard dosimetry laboratory (PSDL). The calibration process consists in the use of a calibration coefficient $N_{D,w}^{{}^{60}\text{Co}}$ (in cGy/C) which converts the measured signal under reference conditions for a given number of MU (in C) to the absorbed dose to water for the same number of MU (in cGy). The conventional reference conditions recommended by TG‐51 are given by a depth in water of 10 cm, a source‐to‐surface distance (SSD) or source‐to‐axis distance (SAD) of 100 cm, and a 10 × 10 cm^2^ square field size defined at the surface for the SSD setup or at the detector for the SAD setup. Since the calibration coefficient is valid for the PSDL's beam quality (i.e., Cobalt‐60, also denoted *Q*_0_), a beam quality correction factor is required to correct the calibration coefficient. The latter is determined using a beam quality specifier, usually $\%dd{\left(10,10\right)}_{X}$, that is, the photon component of a measured percent‐depth dose (PDD) for a 10 × 10 cm^2^ field size and a SSD of 100 cm, at a depth of 10 cm, or TPR_20, 10_(10), that is, the tissue phantom ratio (TPR) in water at depths of 20 cm and 10 cm, for a 10 × 10 cm^2^ field size and a source‐to‐detector distance (SDD) of 100 cm. Equation (1) describes the formalism of TG‐51,^[^ ^1^, ^2^ ^]^

(1) $$D_{w}^{Q}=Mk_{Q}N_{D,w}^{{}^{60}\text{Co}},$$

where $D_{w}^{Q}$ is the absorbed dose to water at the reference depth in the absence of the ionization chamber (in cGy), and $k_{Q}$ is the beam quality correction factor which accounts for the effect of change in beam quality on the chamber dose response between the calibration quality *Q*_0_ (i.e., Cobalt‐60) and the beam quality of the considered linac *Q*. *M* is the chamber reading (in C) corrected for influence quantities other than beam quality, given by^[^ ^2^ ^]^

(2) $$M=P_{\mathrm{TP}}P_{\mathrm{ion}}P_{\mathrm{pol}}P_{\mathrm{elec}}P_{\mathrm{leak}}P_{\mathrm{rp}}M_{\text{raw}}.$$

Note that $P_{\text{TP}}$, $P_{\text{ion}}$, $P_{\text{pol}}$, $P_{\text{elec}}$, and $P_{\text{leak}}$ describe respectively the correction factors for: temperature and pressure, charge recombination, polarity effects, electrometer effects, and leakage. The addendum to TG‐51^[^ ^2^ ^]^ includes the factor $P_{\text{rp}}$ (also known as $P_{\text{vol}}$) which accounts for the radial dose distribution variation over the sensitive volume of the detector (i.e., volume averaging effects).

Since the CyberKnife cannot establish the conventional reference conditions, new msr conditions are introduced in the TRS‐483 for this device. Equation (3) describes the formalism of TRS‐483,^[^ ^3^ ^]^

(3) $$D_{w,Q_{\mathrm{msr}}}^{f_{\mathrm{msr}}}=N_{D,w,Q_{0}}^{f_{\mathrm{ref}}}k_{Q_{\mathrm{msr}},Q_{0}}^{f_{\mathrm{msr}},f_{\mathrm{ref}}}M_{Q_{\mathrm{msr}}}^{f_{\mathrm{msr}}},$$

where $D_{w,Q_{\mathrm{msr}}}^{f_{\mathrm{msr}}}$ is the absorbed dose to water at the msr depth in the absence of the ionization chamber (in cGy), $k_{Q_{\mathrm{msr}},Q_{0}}^{f_{\mathrm{msr}},f_{\mathrm{ref}}}$ is the beam quality correction factor which accounts for the effect of change in beam quality on the chamber dose response between the conventional 10 × 10 cm^2^ reference field of beam quality *Q*_0_ and the msr field of beam quality $Q_{\text{msr}}$, and $M_{Q_{\mathrm{msr}}}^{f_{\mathrm{msr}}}$ is the chamber reading (in C) corrected for influence quantities other than beam quality under msr conditions. $N_{D,w,Q_{0}}^{f_{\mathrm{ref}}}$ is equivalent to $N_{D,w}^{{}^{60}\text{Co}}$ if $Q_{0}\;={\;}^{60}$Co. This notation highlights the fact that this coefficient is valid under the conventional reference conditions (i.e., TG‐51 reference conditions). Note that since $k_{Q_{\mathrm{msr}},Q_{0}}^{f_{\mathrm{msr}},f_{\mathrm{ref}}}$ already includes a correction for volume averaging, the corrected chamber reading described by Equation (2) reduces to

(4) $$M_{Q_{\mathrm{msr}}}^{f_{\mathrm{msr}}}=P_{\mathrm{TP}}P_{\mathrm{ion}}P_{\mathrm{pol}}P_{\mathrm{elec}}P_{\mathrm{leak}}M_{\text{raw}}.$$

Thus, the calibration process can be divided in four main aspects: (1) reference conditions, (2) formalism (i.e., absorbed dose to water determination), (3) beam quality specifier determination, and (4) beam quality correction factor determination. These aspects are summarized in Table 1 for the adapted TG‐51 (i.e., the TG‐51 with manufacturer's adaptations) and the TRS‐483.

**TABLE 1:** Summary of the main aspects of adapted TG‐51 and TRS‐483 reference dosimetry protocols, that is, reference conditions, formalism (i.e., absorbed dose to water determination), beam quality specifier determination, and beam quality correction factor determination. Note that ESFS stands for equivalent square field size.

	Adapted TG‐51	TRS‐483
Reference conditions	Depth in water of 10 cm, circular field size of 6 cm in diameter, SDD of 80 cm	Depth in water of 10 cm, circular field size of 6 cm in diameter, SDD of 80 cm
Formalism	Equations (1) and (2)	Equations (3) and (4) if the beam quality correction factor includes volume averaging effects, or Equations (3) and (2) if it doesn't
Beam quality specifier	$\%dd{\left(10,10\right)}_{X}$ which is inferred using $\%dd{\left(10,6.75\right)}_{X}$, that is, the equivalent value for an ESFS of 6.75 × 6.75 cm^2^, established with the 6 cm collimator	Not needed since beam quality correction factors are tabulated directly for CyberKnife machines
Beam quality correction factor	$k_{Q}=A+B\cdot 10^{-3}\cdot \%dd{\left(10,10\right)}_{X}+C\cdot 10^{-5}\cdot {\left(\%dd{\left(10,10\right)}_{X}\right)}^{2}$ where *A*, *B* and *C* are chamber‐specific fitting parameters provided in the addendum to TG‐51	CyberKnife and chamber‐specific tabulated data in the TRS‐483

Abbreviations: TG, Task Group; TRS, Technical Report Series.

Three situations for the application of TRS‐483 are considered. First, a tabulated beam quality correction factor excluding a generic volume averaging correction is used in conjunction with an in‐house experimentally‐evaluated volume averaging correction factor (i.e., $P_{\text{rp}}$). Second, a tabulated beam quality correction factor including a generic volume averaging correction is used. Third, a MC‐evaluated beam quality correction factor which, by definition, includes a volume averaging correction, is used.

### 2.2 Experimental measurements

**FIGURE 1:**
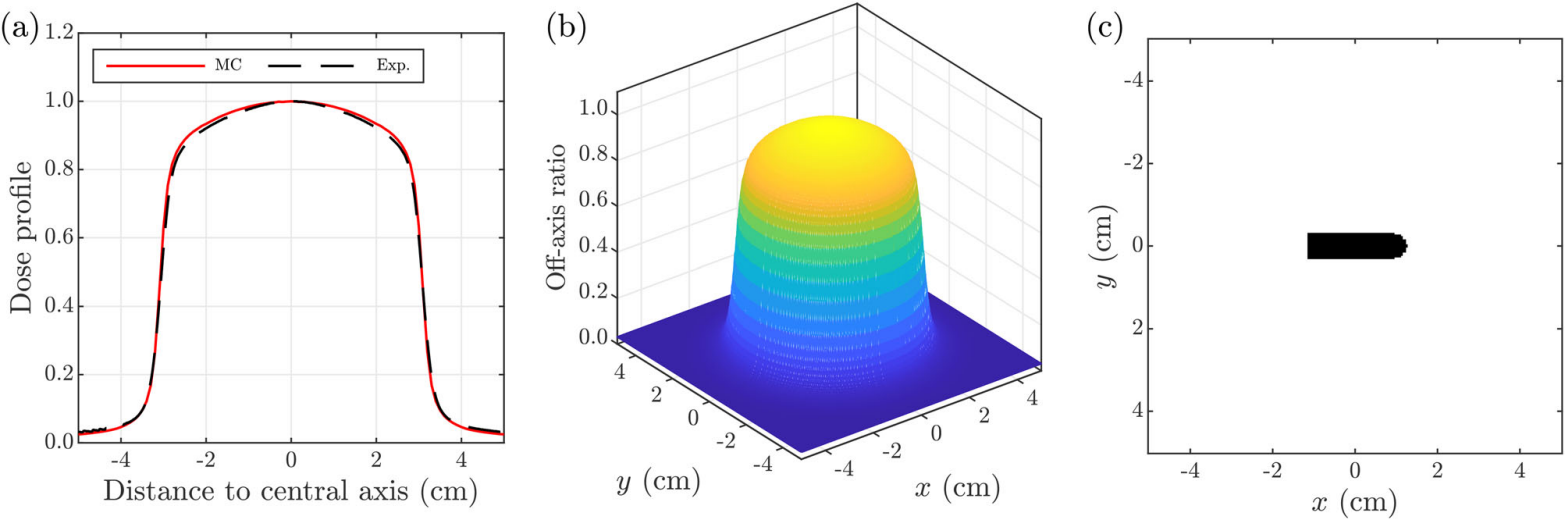
(a) Simulated (i.e., MC) and measured (i.e., experimental) dose profiles for a SSD of 70 cm and a depth in water of 10 cm for the 6 cm collimator of the CyberKnife M6 unit. (b) OAR obtained using the measured dose profile and assuming a cylindrical symmetry for our circular field. (c) Mask representing the dimensions of the sensitive volume of the considered A12 ion chamber. MC, Monte Carlo; OAR, off‐axis ratio; SSD, source‐to surface distance.

Measurements are performed on a CyberKnife M6 unit. All measurements are performed in water using an IBA Blue Phantom^2^ water tank (IBA Dosimetry, Bartlett, Tennessee, USA) and using the 6 cm collimator. For the determination of the beam quality specifier, a PDD is measured using a PTW 60012 diode detector (PTW, Freiburg, Germany) for a SSD of 100 cm. For the determination of the correction factor $P_{\text{rp}}$, a dose profile is measured using a PTW 60012 diode detector, for a SSD of 70 cm and a depth of 10 cm in water. $P_{\text{rp}}$ is estimated by averaging a two‐dimensional off‐axis ratio (OAR) distribution, estimated by assuming a cylindrical symmetry and by using the measured dose profile (see Figure 1a,b), over the dimensions of the sensitive volume of the detector in both directions (see Figure 1c). A calibrated Exradin A12 ionization chamber (Standard Imaging Inc., Middleton, Wisconsin, USA) is used for the reference measurements. The calibration coefficient was obtained from the National Research Council of Canada (NRC) for the considered Exradin A12 chamber and Fluke 35040 (Fluke Biomedical, Everett, Washington, USA) electrometer pair. The reference measurements are performed at a SSD of 70 cm and a depth of 10 cm in water (i.e., a SDD of 80 cm). Measurements performed for the calibration are averaged over five repetitions of 200 MU.

### 2.3 Monte Carlo simulations

Quality correction factors can be evaluated using MC simulations providing the accurate modeling of the considered linac and detector.^[^ ^3^ ^]^ The EGSnrc suite^[^ ^10^, ^11^ ^]^ is used to perform the simulations. The beam quality correction factor introduced in the TRS‐483 can be estimated using

(5) $${\left[k_{Q_{\text{msr}},Q_{0}}^{f_{\text{msr}},f_{\text{ref}}}\right]}_{\text{MC}}=\frac{D_{w,Q_{\text{msr}}}^{f_{\text{msr}}}/{\bar{D}}_{\text{air},Q_{\text{msr}}}^{f_{\text{msr}}}}{D_{w,Q_{0}}^{f_{\text{ref}}}/{\bar{D}}_{\text{air},Q_{0}}^{f_{\text{ref}}}},$$

where $D_{w}$ and ${\bar{D}}_{\text{air}}$ represent the absorbed dose in a small volume of water and the average absorbed dose in the sensitive volume of the considered ionization chamber (i.e., air), respectively. For the former, a small water cylinder with a height of 0.1 cm and a radius of 0.025 cm is used, while for the latter, a fully modeled Exradin A12 ionization chamber, using information of geometry and composition kindly provided by the manufacturer, is used. The subscripts and superscripts $f_{\text{msr}}$ and $Q_{\text{msr}}$ refer to the field size and beam quality under msr conditions. For such simulations, the CyberKnife M6 model developed in Duchaine et al. ^[^ ^12^ ^]^ is used as the source for a 6 cm collimator. The subscripts and superscripts $f_{\text{ref}}$ and *Q*_0_ refer to the field size and beam quality in the chamber calibration conditions (i.e., PSDL's configuration). For such simulations, an isotropic point photon source collimated to obtain a field size of 10 × 10 cm^2^ at a distance of 100 cm is used. The energy spectrum used is the ^60^Co source obtained in Mora et al.^[^ ^13^ ^]^ and provided by default in the EGSnrc code system. Muir and Rogers^[^ ^14^ ^]^ demonstrated that using a tabulated spectrum instead of a full beam model has an impact of less than 0.1% in MC‐evaluated beam quality correction factors.

The volume averaging correction factor in the msr setup can also be estimated through MC simulations using

(6) $${\left[k_{\mathrm{vol}}\right]}_{\text{MC}}=\frac{D_{w,Q_{\mathrm{msr}}}^{f_{\mathrm{msr}}}/{\bar{D}}_{w,\mathrm{vol},Q_{\mathrm{msr}}}^{f_{\mathrm{msr}}}}{D_{w,Q_{0}}^{f_{\mathrm{ref}}}/{\bar{D}}_{w,\mathrm{vol},Q_{0}}^{f_{\mathrm{ref}}}}\approx \frac{D_{w,Q_{\mathrm{msr}}}^{f_{\mathrm{msr}}}}{{\bar{D}}_{w,\mathrm{vol},Q_{\mathrm{msr}}}^{f_{\mathrm{msr}}}},$$

where $D_{w}$ is defined previously and where $D_{\text{w,vol}}$ represents the average absorbed dose in a water volume whose geometry corresponds to that of the sensitive volume of the considered detector (i.e., Exradin A12 ion chamber in this case).

The egs_chamber application^[^ ^15^, ^16^ ^]^ is used to perform the simulations in a 30×30×30 cm^3^ water phantom. The number of histories is set to obtain a statistical uncertainty of 0.1% on the average absorbed dose in the scoring volume.

The dose profile in water at a depth of 10 cm and for a SSD of 70 cm is also simulated in a 30×30×30 cm^3^ water phantom for the 6 cm collimator using the DOSXYZnrc^[^ ^17^ ^]^ application. A spatial resolution of 1×1×1 mm^3^ is used. The number of histories is set to obtain an uncertainty of 0.2% on the average absorbed dose in a single voxel at the center of the beam.

The description of the transport parameters and variance reduction techniques used for the egs_chamber and DOSXYZnrc simulations are summarized in Table 1 of Duchaine et al. ^[^ ^18^ ^]^ The simulation setups of each simulated absorbed dose (i.e., source, field size, SSD, depth, SDD, scoring volume) are summarized in Table 2.

**TABLE 2:** Summary of the simulation setups for each simulated absorbed dose. The 6 cm field size describes a circular field size defined at 80 cm from the CyberKnife source, while the 10 × 10 cm^2^ field size describes the field size at the surface.

Simulated value	Source	Field size	SSD (cm)	Depth (cm)	SDD (cm)	Scoring volume
$D_{w,Q_{\text{msr}}}^{f_{\text{msr}}}$	CyberKnife M6 model	6 cm	70	10	80	Water cylinder
${\bar{D}}_{\text{air},Q_{\text{msr}}}^{f_{\text{msr}}}$	CyberKnife M6 model	6 cm	70	10	80	Exradin A12
$D_{w,Q_{0}}^{f_{\text{ref}}}$	^60^Co source	10 × 10 cm^2^	100	5	105	Water cylinder
${\bar{D}}_{\text{air},Q_{0}}^{f_{\text{ref}}}$	^60^Co source	10 × 10 cm^2^	100	5	105	Exradin A12
${\bar{D}}_{\text{w,vol},Q_{\text{msr}}}^{f_{\text{msr}}}$	CyberKnife M6 model	6 cm	70	10	80	Exradin A12 sensitive volume filled with water
Dose profile	CyberKnife M6 model	6 cm	70	10	−	Water voxels

Abbreviation: SDD, source‐to‐detector distance.

## 3 RESULTS

First, the measured dose profile used for the estimation of $P_{\text{rp}}$ and the simulated dose profile are presented in Figure 1a. Assuming a cylindrical symmetry in our circular field, this results in the two‐dimensional OAR presented in Figure 1b. The latter distribution is obtained using the experimental data. Using the two‐dimensional mask describing the geometry of the sensitive volume of the A12 chamber presented in Figure 1c, this results in an experimental value of $P_{\text{rp}}=1.0107$. Applying the same method to the simulated dose profile, this results in $P_{\text{rp}}=1.0078$.

Then, the MC‐evaluated beam quality correction factor and volume averaging correction factor obtained in this work are respectively ${\left[k_{Q_{\text{msr}},Q_{0}}^{f_{\text{msr}},f_{\text{ref}}}\right]}_{\text{MC}}=1.0004\pm 0.0002$ and ${\left[k_{\text{vol}}\right]}_{\text{MC}}=1.0072\pm 0.0009$, where the reported uncertainties are solely due to beam modeling uncertainties.^[^ ^18^ ^]^

Finally, Tables 3 and 4 summarize the results of the application of the adapted TG‐51 and TRS‐483 protocols (for the three situations described in Section 2.1) for the reference dosimetry our CyberKnife M6 unit. Some parameters, including $N_{D,w,Q_{0}}^{f_{\text{ref}}}$, $P_{\text{TP}}$, $P_{\text{ion}}$, $P_{\text{elec}}$, and $P_{\text{leak}}$, are constant during the application of both protocols. The raw measurements (i.e., $M_{\text{raw}}$/MU) are also constant during the application of both protocols since the reference conditions are the same. The values from which the beam quality specifier $\%dd{\left(10,10\right)}_{X}$ is inferred (i.e., $\%dd{\left(10,\text{ESFS}\right)}_{X}$) are also the same since they represent the same measured value (i.e., PDD measured using the 6 cm collimator for a SSD of 100 cm, at a depth of 10 cm), but are not associated with the same ESFS. The latter parameters are summarized in Table 3. The parameters which vary during the application of both protocols, including the ESFS, beam quality specifier, volume averaging correction factor, beam quality correction factor, and absorbed dose per MU, are found in Table 4 for the three considered situations. The difference between the application of the adapted TG‐51 and the three cases of TRS‐483 are also presented in this table.

**TABLE 3:** Parameters and measurements that are constant between the application of the adapted TG‐51 and TRS‐483.

Parameter	Value
$N_{D,w,Q_{0}}^{f_{\text{ref}}}$	4.87 cGy/nC
$P_{\text{TP}}$	0.9935
$P_{\text{ion}}$	1.0065
$P_{\text{pol}}$	1.0000
$P_{\text{elec}}$	1.0000
$P_{\text{leak}}$	1.0000
$M_{\text{raw}}$/MU	0.1529 nC/MU
$\%dd{\left(10,\text{ESFS}\right)}_{X}$	64.90%

Abbreviations: Carlo; MU, monitor unit; TG, Task Group; TRS, Technical Report Series.

**TABLE 4:** Parameters that are different between the application of the adapted TG‐51 and the TRS‐483.

			TRS‐483	
Parameter	Adapted TG‐51	excluding ${\left[k_{\text{vol}}\right]}_{\text{TRS-483}}$	including ${\left[k_{\text{vol}}\right]}_{\text{TRS-483}}$	using ${\left[k_{Q_{\text{msr}},Q_{0}}^{f_{\text{msr}},f_{\text{ref}}}\right]}_{\text{MC}}$
Formalism	Equations (1) and (2)	Equations (3) and (2)	Equations (3) and (4)	Equations (3) and (4)
ESFS	6.75 × 6.75 cm^2^	Not needed	Not needed	Not needed
$\%dd{\left(10,10\right)}_{X}$	66.94%	Not needed	Not needed	Not needed
$P_{\text{rp}}$	1.0107	1.0107	N.A.	N.A.
$k_{Q}$	$k_{Q}=0.992$	$\frac{{\left[k_{Q_{\text{msr}},Q_{0}}^{f_{\text{msr}},f_{\text{ref}}}\right]}_{\text{TRS-483}}}{{\left[k_{\text{vol}}\right]}_{\text{TRS-483}}}=0.993$ ^a^	${\left[k_{Q_{\text{msr}},Q_{0}}^{f_{\text{msr}},f_{\text{ref}}}\right]}_{\text{TRS-483}}=1.004$ ^b^	${\left[k_{Q_{\text{msr}},Q_{0}}^{f_{\text{msr}},f_{\text{ref}}}\right]}_{\text{MC}}=1.0004$ ^c^
$D_{w,Q_{\text{msr}}}^{f_{\text{msr}}}$/MU	0.746 cGy/MU	0.747 cGy/MU	0.748 cGy/MU	0.7449 cGy/MU
Diff. with adapted TG‐51	−	+0.11%	+0.14%	−0.22%

^a^ Generic quality correction factor provided in tab. 33 of TRS‐483 and excluding the generic volume averaging correction of ${\left[k_{\text{vol}}\right]}_{\text{TRS-483}}=1.011$.

^b^ Generic quality correction factor provided in tab. 13 of TRS‐483 and including the generic volume averaging correction of ${\left[k_{\text{vol}}\right]}_{\text{TRS-483}}=1.011$.

^c^ MC‐evaluated beam quality correction factor that includes a volume averaging correction of ${\left[k_{\text{vol}}\right]}_{\text{MC}}=1.0072$.

Abbreviations: MC, Monte Carlo; MU, monitor unit; TG, Task Group; TRS, Technical Report Series.

## 4 DISCUSSION

The first difference observed between the application of both protocols is the ESFS of the 6 cm collimator of the CyberKnife. While the manufacturer recommends an ESFS of 6.75× 6.75 cm^2^ for this collimator for a SSD of 100 cm, the TRS‐483 estimates a value of 5× 5 cm^2^ at a distance of 80 cm from the source, which results in an ESFS of 6.25× 6.25 cm^2^ when projected to a distance of 100 cm, due to the presence of steeper gradients in this field (see tab. 16 of TRS‐483). In combination with the fact that different methods are used for the conversion of $\%dd{\left(10,\text{ESFS}\right)}_{X}$ to $\%dd{\left(10,10\right)}_{X}$, this results in a value of $\%dd{\left(10,10\right)}_{X}=67.42\%$ for the TRS‐483 protocol, which differs by 0.71% from the adapted TG‐51 protocol. It is interesting to note that according to the addendum to TG‐51, a change in 1% in $\%dd{\left(10,10\right)}_{X}$ leads to a 0.15% change in the beam quality correction factor.^[^ ^2^ ^]^ Thus, the difference of 0.71% in beam quality specifier observed in this study should have an impact of less than 0.15% on the quality correction factor. However, since, in TRS‐483, the latter are tabulated for the CyberKnife (see tabs. 13 and 33 of TRS‐483) and are not a function of the beam quality specifier per se, the estimated value of $\%dd{\left(10,10\right)}_{X}=67.42\%$ is not used directly in the protocol.

Furthermore, the obtained MC‐evaluated beam quality correction factor (i.e., ${\left[k_{Q_{\text{msr}},Q_{0}}^{f_{\text{msr}},f_{\text{ref}}}\right]}_{\text{MC}}=1.0004\pm 0.0002$) can be compared to the TRS‐483 tabulated value for the CyberKnife and the Exradin A12 chamber including a generic correction factor for volume averaging effects (i.e., ${\left[k_{Q_{\text{msr}},Q_{0}}^{f_{\text{msr}},f_{\text{ref}}}\right]}_{\text{TRS-483}}=1.004\pm 0.010$). A difference of 0.36% is observed. This is attributed to disparities in volume averaging effect corrections. Indeed, a difference of 0.38% is observed between $k_{\text{vol}}$ obtained in this work using MC simulations (i.e., ${\left[k_{\text{vol}}\right]}_{\text{MC}}=1.0072\pm 0.0009$) and the value estimated by TRS‐483 (i.e., ${\left[k_{\text{vol}}\right]}_{\text{TRS-483}}=1.011$). ${\left[k_{\text{vol}}\right]}_{\text{MC}}$ is smaller than the value estimated experimentally (i.e., $P_{\text{rp}}=1.0107$) which is explained by the fact that the simulated beam profile is slightly flatter than the measured one as shown in Figure 1a (i.e., the analytical results for $P_{\text{rp}}$ with the simulated profile is 1.0078). The comparison of the $k_{Q_{\text{msr}},Q_{0}}^{f_{\text{msr}},f_{\text{ref}}}$ values to which the respective contribution of averaging effects is removed (i.e., ${\left[k_{Q_{\text{msr}},Q_{0}}^{f_{\text{msr}},f_{\text{ref}}}\right]}_{\text{TRS-483}}/{\left[k_{\text{vol}}\right]}_{\text{TRS-483}}=0.993$ and ${\left[k_{Q_{\text{msr}},Q_{0}}^{f_{\text{msr}},f_{\text{ref}}}\right]}_{\text{MC}}/{\left[k_{\text{vol}}\right]}_{\text{MC}}=0.9932$) illustrates a reduction of the difference to 0.02%. This is consistent with the results of Francescon et al. ^[^ ^19^ ^]^ The authors observed that $k_{Q_{\text{msr}},Q_{0}}^{f_{\text{msr}},f_{\text{ref}}}$ is 0.4% lower for the CyberKnife M6 compared to previous versions of the CyberKnife (G3, G4, and VSI, that is, versions on which the TRS‐483 based its recommendations for $k_{\text{vol}}$) for Farmer type chambers since the dose profile is more uniform over the sensitive volume of the chambers for the CyberKnife M6. Francescon et al. ^[^ ^19^ ^]^ reported a value of $k_{Q_{\text{msr}},Q_{0}}^{f_{\text{msr}},f_{\text{ref}}}=1.0000\pm 0.0016$ for the Exradin A12 chamber and the CyberKnife M6, which is in agreement with the value obtained in this work with an observed difference of 0.04%. The difference in volume averaging correction factors is also causing the observed difference between the adapted TG‐51 and the TRS‐483, and between the three situations themselves. However, the discrepancies in absorbed dose to water per MU in the calibration of the CyberKnife M6 using the adapted TG‐51 protocol versus the TRS‐483 protocol for the three cases considered are not statistically significant considering that an uncertainty of 1% is estimated for the beam quality correction factors provided in TRS‐483. Thus, the TRS‐483 protocol is found to be consistent with the adapted TG‐51 protocol.

The fact that the TRS‐483 protocol is consistent with the adapted TG‐51 protocol is attributed to the fact that, by design, both formalisms are similar since the formalism introduced in the TRS‐483 reduces to the TG‐51 if conventional reference conditions can be established, and to the use of the same reference conditions in this case. Indeed, the recommended reference conditions of the adapted TG‐51 and the TRS‐483 (i.e., a circular field size of 6 cm in diameter defined at a SDD of 80 cm, and a depth in water of 10 cm) are identical. Results illustrate that the difference in terms of absorbed dose is minimized when the same volume averaging correction factor is used for the application of both protocols. Overall, the results obtained in this study are consistent with results found in literature^[^ ^5^, ^6^, ^7^, ^8^ ^]^ and summarized in Section 1.

In terms of workload, no significant difference is observed for the application of both protocols. The main source of work is the implementation of a new protocol in a clinical context which requires a complete understanding of the recommendations of the TRS‐483, which is non‐negligible. However, the measurements required to perform the calibration are comparable (i.e., determination of the beam quality specifier using $\%dd{\left(10,\text{ESFS}\right)}_{X}$ measurements, determination of in‐house volume averaging correction factor with dose profile measurements, and measurements using the calibrated chamber under msr conditions). The conversion of $\%dd{\left(10,\text{ESFS}\right)}_{X}$ to $\%dd{\left(10,10\right)}_{X}$ is simpler in TRS‐483 as a simple equation is provided (eq. 29 of TRS‐483). Tabulated beam quality correction factors for the CyberKnife and many chambers are provided, which renders the determination of the beam quality specifier a verification process. One should however be careful when using the tabulated data since they include generic volume averaging correction factors which might be outdated in the case of the CyberKnife M6. A procedure is provided in TRS‐483 to convert tabulated beam quality correction factor which includes a generic volume averaging correction, for use with an in‐house (or specific) volume averaging correction (eq. 57 of TRS‐483). Once again, as the formalism of TRS‐483 was designed to reduce to the formalism of conventional dosimetry protocols (e.g., TG‐51) when conventional reference conditions can be established, it is not surprising that the difference in workload is minimal once the new protocol is implemented.

The main limitation of this study is the detector used, which is a Farmer type ionization chamber. The latter are considered too large for the reference dosimetry of ESFS between 3× 3 cm^2^ and 5× 5 cm^2^ in 6 MV beams even if lateral charged particle equilibrium (LCPE) exists due to significant volume averaging issues.^[^ ^3^ ^]^ In our case, LCPE conditions are respected, but the ESFS of the 6 cm collimator at a distance of 80 cm from the source is 5× 5 cm^2^, which is at the limit of what is recommended. However, some studies argue that the use of Farmer chambers in msr fields is not problematic, as long as volume averaging is taken into account.^[^ ^20^, ^21^ ^]^

## 5 CONCLUSION

An adapted version of the TG‐51 protocol and the TRS‐483 protocol are applied for the calibration of a CyberKnife M6 unit using an Exradin A12 ionization chamber. The main source of discrepancy in calibration when an in‐house volume averaging correction is used is the difference in beam quality correction factors. This induces a difference of 0.11% in absorbed dose to water per MU. This difference slightly increases to 0.14% when a generic volume averaging correction factor is used for the TRS‐483 application. MC results illustrate that the generic beam quality correction factor provided in TRS‐483 might be overestimated by 0.36% compared to our specific model and that this overestimation could be due to the volume averaging component. In all cases, the discrepancy is considered non‐statistically significant considering the reported uncertainty on the tabulated value of beam quality correction factor reported in the TRS‐483. Thus, for clinical reference dosimetry of the CyberKnife M6, the application of IAEA's TRS‐483 is found to be consistent with AAPM'S TG‐51, while differences in terms of workload are found to be insignificant.

## AUTHOR CONTRIBUTIONS

*Development of the original idea; Digital development with Matlab; Geometric modeling of the detector with egs_chamber; Numerical Monte Carlo calculations with EGSnrc; Analysis of numerical and experimental results; Writing of the paper*: Jasmine Duchaine. *Experimental measurements; General discussion of the project; Paper revision*: Daniel Markel. *Experimental measurements; General discussion of the project; Paper revision*: Jean‐Luc Ley. *Experimental measurements; General discussion of the project; Paper revision*: Dominic Béliveau‐Nadeau. *Experimental measurements; General discussion of the project; Paper revision*: Karim Zerouali. *Experimental measurements; General discussion of the project; Paper revision*: Robert Doucet. *Project management and scientific support; Development of the original idea; Paper revision*: Hugo Bouchard.

## CONFLICT OF INTEREST STATEMENT

The authors have no conflicts to disclose.

## Data Availability

The data that support the findings of this study are available from the corresponding author upon request.
